# It's No Fluke: The Planarian as a Model for Understanding Schistosomes

**DOI:** 10.1371/journal.ppat.1003396

**Published:** 2013-07-18

**Authors:** James J. Collins, Phillip A. Newmark

**Affiliations:** Howard Hughes Medical Institute and Department of Cell and Developmental Biology, Neuroscience Program, University of Illinois at Urbana-Champaign, Urbana, Illinois, United States of America; University of Wisconsin Medical School, United States of America

## Introduction

Schistosomiasis is among the most prevalent human parasitic diseases, affecting more than 200 million people in the developing world [1]. Indeed, some estimates suggest the global disease-associated disability resulting from schistosomiasis may reach levels on par with global killers including malaria, tuberculosis, or HIV/AIDS [2], [3]. The causative agents of this disease are flatworms (*Schistosoma* or blood flukes) that feed on blood in the veins surrounding the host's intestine or bladder. These worms produce hundreds to thousands of eggs per day, many of which lodge in host tissues and cause diverse pathologies, including hepatic fibrosis, splenomegaly, and in some cases, perhaps cancer. Despite the grim statistics, only a single therapeutic agent (praziquantel) is currently used to treat schistosome infection. With the looming possibility that praziquantel-resistant *Schistosoma* strains could arise, an urgent need exists to identify novel therapeutic agents to combat these parasites.

## Schistosomes Have Complicated Life Cycles

**Figure 1:**
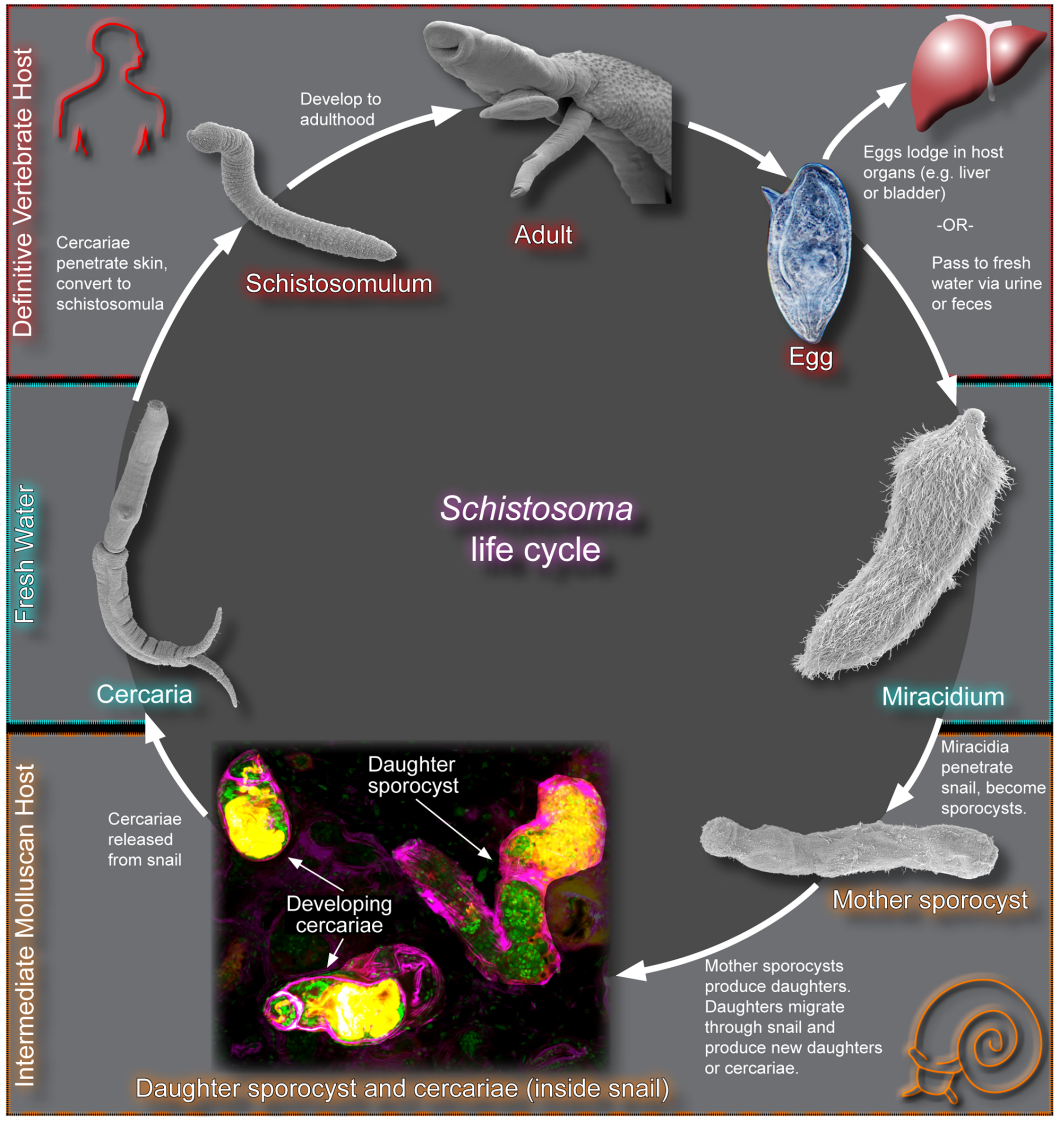
The schistosome life cycle. The various stages of the schistosome life cycle are depicted using fluorescence (sporocysts and cercariae in snail host labeled with DAPI (green), phalloidin (magenta), and peanut agglutinin (orange)), bright-field (egg), or scanning electron micrographs (remaining stages). Images are not shown to scale.

Like all digenetic trematodes, schistosomes have complex life cycles that alternate between phases of obligate parasitism of snail intermediate and vertebrate definitive hosts [4] (Figure 1). This life cycle initiates when schistosome eggs, passed via the urine or feces of a vertebrate host, come into contact with freshwater and release a short-lived, ciliated stage called the miracidium. These miracidia employ a sophisticated set of sensory and penetration organs to seek out and ultimately infect a suitable snail host. Following penetration, miracidia undergo a striking developmental transformation whereby major organ systems degenerate and pluripotent cells called germinal cells begin to proliferate. This stage of the life cycle, the sporocyst, is key to the successful propagation of schistosomes. Simply put, stem cells in the sporocysts have the capacity to undergo an asexual embryogenesis, producing either additional sporocysts or thousands of infective cercaria, which are released from the snail. Attracted by fatty acids from vertebrate skin, cercariae burrow through the host's epidermis, enter the circulatory system, and eventually migrate to the mesenteric veins (*S. mansoni* and *S. japonicum*) or the venous plexus of the bladder (S. *haematobium*). The parasites then feed on blood, develop to adulthood as either male or female worms (which then pair together), and begin to reproduce, thus completing the life cycle.

Over the past decade, a wealth of modern molecular tools have been made available to study schistosomes, including: genome sequences for the three major human schistosome species [5]–[7], procedures for genetic manipulation using RNA interference [8], [9], robust protocols for whole-mount in situ hybridization [10] and immunofluorescence [11], and breakthroughs in the development of transgenic approaches [12]. However, the complexity of the schistosome life cycle, coupled with a dearth of methods for *in vitro* propagation, has continued to hinder progress toward developing novel therapeutics. Therefore, new approaches are needed to address this important challenge.

## Lessons from Roundworms

Studies of model organisms (e.g., yeast, ciliated protozoans, nematode worms, and fruit flies) have shaped our understanding of central questions in biology (e.g., cell-cycle control, telomerase function, programmed cell death, and embryonic patterning). Similarly, free-living invertebrates have provided meaningful contributions toward our understanding of their parasitic relatives. For example, studies of the free-living nematode *Caenorhabditis elegans* have provided novel insight into nematicidal drug action [13]–[15], parasite genome evolution [16], and parasite gene function [17]–[19]. Procedures developed for *C. elegans* have even guided the establishment of transgenic approaches for parasitic nematodes [20]. Furthermore, studies of *C. elegans* have revealed how exploiting “eccentricities” shared between free-living and parasitic nematodes could lead to novel ways to combat disease. For example, a nuclear hormone receptor (DAF-12) that regulates a *C. elegans* alternative third-larval stage (L3), called Dauer, similarly regulates the analogous infective L3 (iL3) stage of parasitic nematodes [21]. Of note, administration of the *C. elegans* DAF-12 ligand is capable of inducing precocious iL3 recovery of *Strongyloides stercoralis* or *Ancylostoma caninum* and causing *S. stercoralis* to aberrantly enter iL3. Thus, the modulation of DAF-12 activity represents a novel means to disrupt the life cycle of parasitic nematodes.

**Figure 2:**
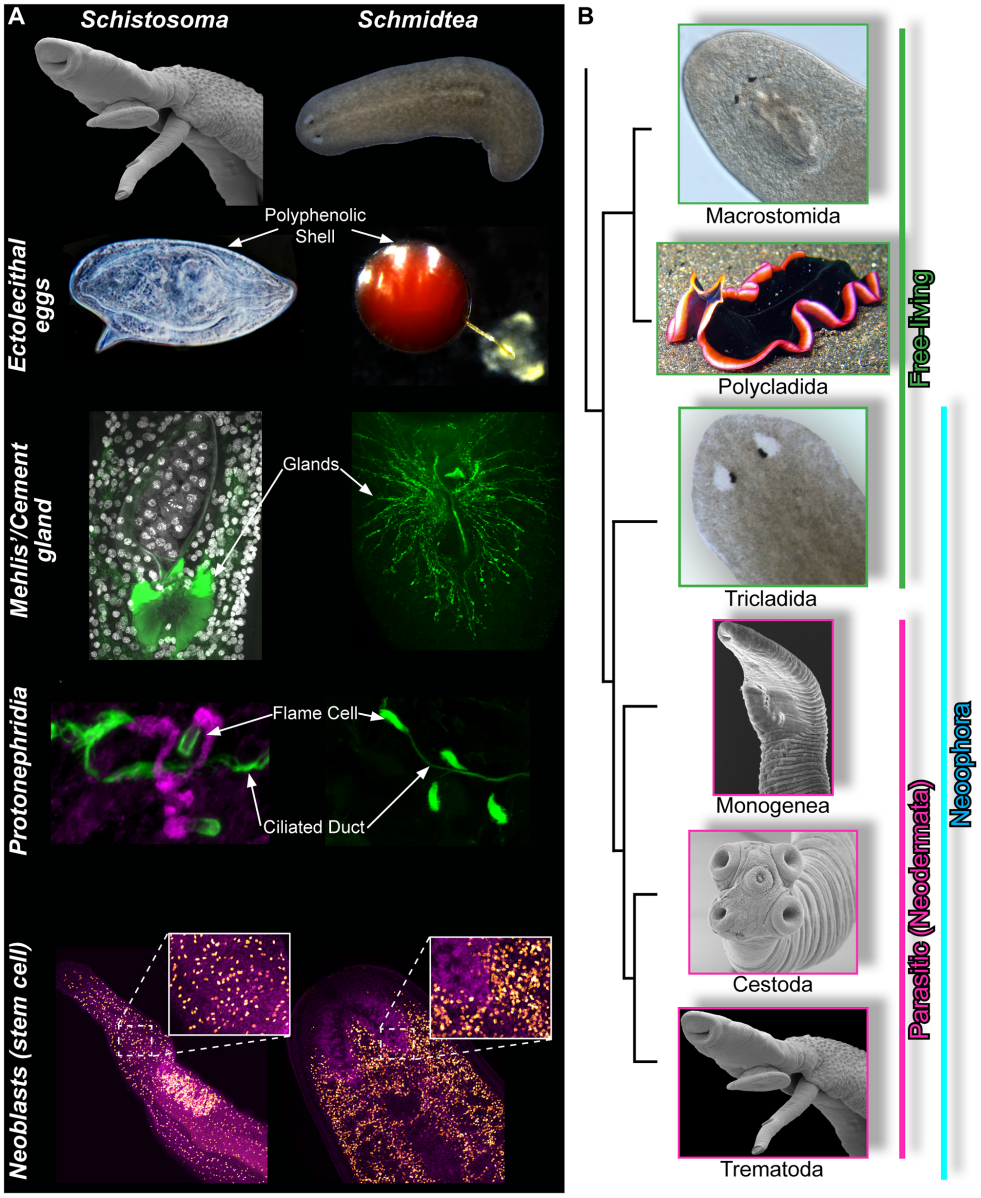
Similarities between schistosomes and planarians. (A) Schistosomes (left column) and planarians (right column) possess common cell types and organ systems. Top row, (left) scanning electron micrograph of male and female *S. mansoni* and (right) dark-field image of *S. mediterranea*. Second row, eggs. Both organisms lay ectolecithal egg capsules with a polyphenolic shell. Only a subset of derived flatworm species (called neoophora) lay this unique type of egg. Third row, glands associated with the egg-laying apparatus. These glands (Mehlis' gland in schistosomes and cement gland in planarians) labeled with the lectin, peanut agglutinin (green) [11], [37]. Although these glands are found in both free-living and parasitic flatworms, their function is unclear. Fourth row, confocal images of protonephridia labeled with (left) anti-acetylated tubulin (green)/wheat germ agglutinin (magenta) and (right) anti-acetylated tubulin (green). Protonephridia are found in most platyhelminths and play essential roles in controlling water balance [38], [39]. Since schistosomes conform to the osmolarity of their host, it is also likely this organ system has additional functions (e.g., excreting waste). Fifth row, labeling with thymidine analogs to detect neoblasts (orange nuclei). All other nuclei are magenta. (B) Simplified phylogeny of the Platyhelminthes. Images depict representative genera from each group. Images from each group are as follows: Macrostomida (*Macrostomum* sp.), Polycladida (*Pseudobiceros hancockanus*, credit: J. Petersen), Tricladida (*Schmidtea mediterranea*), Monogenea (*Diplozoon* sp., credit: D. Thenet, R. Kirk, and P. Olson), Cestoda (*Hymenolepis* sp., credit: P. Olson), Trematoda (*Schistosoma mansoni*).

Although work on *C. elegans* has contributed to our understanding of nematode parasites, no equivalent free-living models have become widely accepted for studies of the phylogenetically distinct parasitic flatworms (e.g., *Schistosoma* and *Fasciola*). Indeed, not all worms are created equal: nematodes are members of a group of molting animals called Ecdysozoa, whereas flatworms are Lophotrochozoans, a clade of animals that includes mollusks and annelids [22]. Thus, in evolutionary terms, schistosomes share more in common with snails and squid than they do with hookworms and whipworms! Despite their differing lifestyles, free-living flatworms and their parasitic relatives share numerous evolutionarily conserved features that are central to their physiology and reproduction (a few examples are shown in Figure 2A and are discussed below). Furthermore, in contrast to the nematodes, in which parasitism has arisen multiple times independently [23], all parasitic flatworms (trematodes, cestodes, and monogeneans) form a derived monophyletic group known as the “neodermata” (Figure 2B). Thus, applying lessons learned from free-living relatives has the potential to directly aid our understanding of not just schistosomes, but the more than 6,000 species of parasitic flatworms.

## Planarians as a Free-Living Model for Schistosomes?

Planarians are free-living platyhelminths and have been a valued experimental model for over a century due to their nearly unlimited capacity to regenerate damaged tissues [24]. Indeed, the regenerative abilities of planarians are so robust that minuscule tissue fragments (<1/200^th^ the size of the original animal) can fully regenerate a new animal. This regenerative prowess is made possible by a population of pluripotent stem cells, called neoblasts, which proliferate and serve as progenitors for regenerating tissues. In addition to their role in regeneration, neoblasts also drive long-term tissue homeostasis such that planarians deprived of neoblasts fail to maintain their tissues and die within a few weeks.

Over the past decade, *Schmidtea mediterranea* has become the preeminent model for understanding the biology of stem cells and regeneration in planarians. Its rise to prominence is due in part to the ease with which it can be maintained in the laboratory, its favorable genetic features (i.e., relatively small diploid genome), and its fully sequenced and assembled genome [24], [25]. Furthermore, robust methods exist for analysis of gene function, including the ability to perform large-scale whole-mount in situ hybridization screens and to perturb the function of hundreds to thousands of genes by simply feeding animals gene-specific dsRNAs [24], [26]. As studies of parasitic and free-living nematodes would suggest, taking advantage of these experimental attributes may allow researchers to reveal peculiarities common to free-living and parasitic flatworms that could be exploited in the treatment of disease.

## Schistosomiasis: A Disease of Flatworm Reproduction

The pathology associated with schistosome infection is almost entirely due to egg-induced inflammation. Thus, schistosomiasis can be thought of as disease of flatworm reproduction, and blunting the prodigious schistosome reproductive “machine” could have tremendous therapeutic potential. Although schistosomes are dioecious (having separate male and female sexes) and planarians are simultaneous hermaphrodites (containing both male and female reproductive organs in one animal), both the arrangement of the plumbing (i.e., the ducts connecting various organs) and the major components (e.g., yolk glands and glands for eggshell production) of the male and female reproductive systems are conserved [27] (Figure 2A). Furthermore, both planarians and schistosomes are bizarre (even by flatworm standards!) in that they produce ectolecithal eggs—in which specialized yolk cells surround the fertilized egg before being packed into the eggshell. As a result, both planarians and schistosomes have dedicated organs for producing these yolk cells: the vitellaria in schistosomes and the yolk glands in planarians. Highlighting the potential for planarians to aid in our understanding of schistosome reproduction, a study of planarian spermatogenesis identified genes specific to flatworms that were required for normal planarian sperm production [28]. Expanding on this type of work to study other processes (e.g., oogenesis, yolk production, egg-capsule formation) is likely to identify additional factors that regulate the rather unusual aspects of the flatworm reproductive system. Exploiting such “quirks” could ultimately be fertile ground for therapeutic intervention.

A truly fascinating feature of schistosome biology is that the female reproductive system is extremely plastic, and its development and maintenance require constant pairing with a male worm. In particular, the reproductive organs of females from single-sex infections are developmentally stunted and only initiate development when pairing with a male is established [4]. Similarly, the reproductive organs of mature females separated from their male partner are resorbed and are only able to regenerate once male-female pairing is reestablished. Although this intriguing biological phenomenon has confounded scientists for decades, studies of planarians may offer unique clues for understanding how this plasticity is regulated. Similar to schistosomes, planarians can also resorb and regenerate their reproductive system in response to a variety of cues, including body size, injury, nutrition, and season [24], [29], [30]. One study aimed at understanding this reproductive plasticity in planarians uncovered a neuroendocrine factor, called *npy-8*, that was essential for maintaining the reproductive system of adult planarians [31]. This work showed that *npy-8* was expressed exclusively in the nervous system of sexually reproducing planarians and that abrogation of *npy-8* function resulted in regression of the reproductive organs in adult planarians. Since an ortholog of *npy-8* is found in the genome of *Schistosoma mansoni*, it is tantalizing to speculate that planarians and schistosomes may use similar neuroendocrine mechanisms to control the development of their reproductive systems. As studies continue to define the biochemical milieu that promotes schistosome reproduction [32], suitable *in vitro* conditions may one day exist to experimentally assess the function of this *npy-8*–like gene in schistosome reproduction.

## Like Planarians, Schistosomes Have Neoblasts

Although the eggs are the source of pathology in schistosomiasis, the chronic nature of this disease can be attributed in part to the extreme longevity of these parasites. In fact, reproductively active schistosomes have been observed in patients more than three decades after leaving endemic regions [4]. While the mechanisms responsible for this longevity remain a mystery, studies of planarians could provide new insights. As mentioned above, neoblasts not only fuel regeneration but they also promote the renewal of tissues lost during the normal wear and tear of daily life. Guided by this knowledge, and the observation that neoblast-like cells have been described in tapeworms [33], a recent study identified a novel population of neoblast-like stem cells in adult *S. mansoni* [34]. These cells were morphologically similar to neoblasts and also shared their ability to produce differentiated cell types, such as muscle and intestine. Furthermore, these cells expressed numerous genes known to regulate neoblasts in planarians. At present, it is not clear what role these cells play in the parasite; however, the observation that schistosomes can regenerate damaged tissues following sublethal doses of praziquantel [35] suggests that these stem cells may play a pivotal role, not only in longevity, but in numerous facets of the parasite's biology.

## Conclusions

This short pearl has only scratched the surface of the potential for using planarians to inform the biology of schistosomes. Indeed, many questions remain: Can planarians be used to identify new therapeutics? Can planarians help us characterize the mechanism of action of existing therapeutics [36]? Are any of the hundreds of genes specific to flatworms functionally important? Do neoblast-like stem cells play a role in other stages of the schistosome life cycle?

Due to space constraints, this review has focused upon features shared between these flatworms; however, it is important to note that there are fundamental differences between them, too. Schistosomes must invade two different hosts, navigate their immune systems, and respond to (and in some cases modulate) host physiology. For all that we may learn about flatworm biology by studying planarians, it remains absolutely critical to study the unique biology of schistosomes. Research capitalizing on the genomic resources available for these parasites, and applying the functional tools used so successfully to study planarians, is expected to open new frontiers for understanding schistosomes and for reducing the burden of the disease they cause.
